# An eye-tracking study of visual attention in chimpanzees and bonobos when viewing different tool-using techniques

**DOI:** 10.1007/s10071-025-01934-5

**Published:** 2025-02-11

**Authors:** Yige Piao, James Brooks, Shinya Yamamoto

**Affiliations:** 1 Wildlife Research Center, Kyoto University, Kyoto, Japan; 2 Institute for Advanced Study, Kyoto University, Kyoto, Japan; 3 German Primate Center (DPZ), Göttingen, Germany; 4 Institute for the Future of Human Society, Kyoto University, Kyoto, Japan

**Keywords:** Tool-using technique, Social learning, Eye-tracking, Visual attention, Chimpanzee, Bonobo

## Abstract

Chimpanzees and bonobos are excellent tool users and can socially learn various skills. Previous studies on social learning mainly measure success/failure in acquiring new techniques, with less direct measurement of proximate mechanisms like visual attention during the process. This study investigates how great apes observe tool-using demonstrations through eye-tracking. After checking initial techniques, six chimpanzees and six bonobos were shown video demonstrations of human demonstrators using a tube to dip (low-efficiency) or suck (high-efficiency) juice, and then tried the task themselves. Attention to each video was compared to participants’ knowledge. Although no individuals acquired the high-efficiency technique through video demonstrations, eye-tracking results revealed attentional differences between individuals familiar with different techniques. Compared with individuals already familiar with both techniques, individuals knowing only the dipping technique showed less attention to the unfamiliar sucking technique. This result indicates that apes may not attend much to what they do not know well, which aligns with reported interplay of action observation and understanding. Attentional patterns to the action part of the two techniques was non-significant between species, though bonobos looked marginally more at faces and chimpanzees looked significantly more at food. This study highlights the importance of conducting detailed investigations into social learning processes, with eye-tracking as one valuable method.

**Supplementary Information:**

The online version contains supplementary material available at 10.1007/s10071-025-01934-5.

## Introduction

Chimpanzees and bonobos, our evolutionary closest relatives, stand out among non-human tool-users in both repertoire and ability to socially learn (Whiten and van de Waal 2018), providing significant insight into the evolution of human culture (Whiten 2005; Whiten 2017; Whiten and van de Waal 2017). Wild chimpanzees demonstrate an impressive array of tool-use, including underground termite fishing and nut-cracking (Boesch and Boesch 1983; Matsuzawa 2011; Sanz et al. 2004; Suzuki et al. 1995), in their material culture (Boesch and Tomasello 1998; Mcgrew 1992; Whiten et al. 1999). While wild bonobos seldom use tools (Furuichi et al. 2015; Gruber and Clay 2016), bonobos in captivity are as skilled at tool-use as chimpanzees (Neufuss et al. 2017; Roffman et al. 2012, 2015). Though individuals may innovate behaviors through asocial learning (Bandini and Tennie 2020), social learning is pivotal in the transmission and maintenance of such behaviors (Hobaiter et al. 2014; Tomasello et al. 1987; van Leeuwen et al. 2020; Whiten and van de Waal 2018) and even leads to local traditions and cultural variations (Hohmann and Fruth 2003; Hopper et al. 2007; van Schaik et al. 2003; Whiten et al. 2007).

Over the decades, social learning of tool-use has been a hot topic in cognitive studies of great apes (van Schaik and Pradhan 2003; Whiten 2000; Whiten and van de Waal 2018). However, previous studies mostly focused on the success/failure in acquiring new skills and the transmission network (Franz and Nunn 2009; Horner et al. 2006; Marshall-Pescini and Whiten 2008; Vale et al. 2017), while the underpinning mechanisms remain underexplored. More specifically, little is known about how observers view demonstrations during this process. As social learning involves obtaining information from other individuals (Heyes 2012), investigating this information extraction process is essential to fully understand social learning and its role in facilitating traditions and cultures.

Eye-tracking technology provides qualitative and quantitative investigation into the cognitive process of non-human primates (Hopper et al. 2020). Previous eye-tracking studies have indicated some differences between chimpanzees and bonobos, like species-specific viewing patterns (Kano et al. 2015), sex-based social attention (Lewis et al. 2021), and social attention influenced by oxytocin (Brooks et al. 2021). But the information about their similarities or differences in the attention to or the ability to learn from tool-using videos is scarce. Here we investigate how *Pan* species view demonstrations of familiar and unfamiliar tool-using techniques. We adopted a simple tool-using task used in a previous study (Yamamoto et al. 2013). Apes could use a transparent tube to obtain grape juice through a small hole. The tube could be used as either a stick to dip (low-efficiency) or a straw to suck (high-efficiency). Within a group, multiple techniques may be innovated based on individual experience and knowledge, providing the opportunity to investigate how social attention to differing techniques is affected by prior knowledge (Laland 2004; Rendell et al. 2011).

We first checked participants’ initial tube-using techniques and then used eye-tracking technology to investigate their visual attention when observing human demonstrators displaying the two solutions that differ in efficiency. We predicted that visual attention would differ between individuals with differing initial techniques and between bonobos and chimpanzees. More specifically, previous studies indicated a close link between action observing, understanding, and execution (Kanakogi and Itakura 2010; Liepelt et al. 2008). Therefore, our first hypothesis was: (1) visual attention would differ between participants with different baseline techniques, especially regarding the more challenging high-efficiency sucking technique. Based on the attentional and motivational differences between the species (Herrmann et al. 2010; Kano et al. 2015), our second hypothesis was: (2) chimpanzees would look more at the action part (i.e. tube and hand), while bonobos would look more at demonstrators’ face. As the key differences between the two techniques are mainly displayed in the action part, if the second hypothesis is supported, we would expect dipping-technique chimpanzees to pay close attention to the action part and notice the differences between two techniques, leading to higher chances of learning the new sucking technique. Dipping-technique bonobos, however, may pay more attention to the face part and fail to notice the differences displayed in the action part, leading to the possible failure of learning the sucking technique. Therefore, the third hypothesis was: (3) dipping-technique chimpanzees would be more likely than dipping-technique bonobos to notice the different technique and switch to the higher-efficiency sucking method. The predicted “switch” result is specific to dipping-technique participants, because a previous study (Yamamoto et al. 2013) demonstrated that dipping-technique (low-efficiency) participants switched to sucking (high-efficiency) by social observation, but not vice versa.

## Methods

### Animals

Six chimpanzees (*Pan troglodytes verus*) and six bonobos (*Pan paniscus*) at Kumamoto Sanctuary, Japan, participated in this study (see details in supplementary materials).

Prior to the experiment, participants were offered several transparent plastic tubes (diameter: inner 4 mm, outer 7 mm; length around 23 cm) and a bottle of grape juice (inner diameter 4 cm, height 19 cm). The plastic tube could be used as a tool to either dip (low-efficiency) or suck (high-efficiency) the juice. Participants’ initial methods were recorded as their knowledge of tube-using techniques.

### Eye-tracking setup

The procedures were similar to previous studies (Brooks et al. 2021; Kano et al. 2015). An infrared eye tracker (300 Hz, TX300, Tobii Technology AB) was used to record the apes’ visual attention. A 23-inch LCD monitor (43 × 24°, resolution 1280 × 720 pixels) displayed the stimuli from a distance of 60 cm to the participant. During eye-tracking, the participant was continuously provided with grape juice drips via a custom-made juice dispenser. Automated calibration was performed for each participant by presenting a video clip on two reference points, and the result was shown on the monitor by presenting small reference icons. This calibration was repeated whenever necessary before each eye-tracking recording. Calibration errors are generally within one degree for most participants (Kano et al. 2011).

Visual stimuli were two videos in which two human experimenters displayed either dipping or sucking techniques (Video S1). All participants were familiar with both experimenters for over seven months (Y.P.) to four years (J.B.). Each video lasted 24 s, with the first demonstrator (J.B.) performing the action four times in 12 s and the second demonstrator (Y.P.) doing the same in another 12 s. In dipping-technique videos, demonstrators dipped one end of the tube into the juice through a hole (diameter 1.2 cm) on the panel, retrieved the tube, and put that end into the mouth. In sucking-technique videos, demonstrators put one end of the tube into the juice through the hole and sucked some juice via the other end. The juice was completely visible when moving inside the tube, and the liquid level in the bottle visibly decreased.

### General procedure

In each trial, participants were called by their names, and they could freely choose to enter the testing room or not. The testing room for chimpanzees is a square room measuring 3.3 m in length, 3.3 m in width, and 2.05 m in height. The testing room for bonobos is a rectangular room (5.2 (L) × 3.0 (W) × 3.3 (H) m). The panel through which participants were eye-tracked was made of acrylic (1 cm thickness).

Trials began when a participant voluntarily approached the panel and was shown the juice bottle and tube. The participant then watched the video demonstrations while sucking on the juice drip, and her/his gaze was recorded via eye-tracking. After the demonstrations, the experimenter again showed the juice bottle and added some juice into it. Finally, a tube was provided through the hole on the panel, and the juice bottle was attached below this hole using a suction cup (diameter 25 mm). The participant was offered one minute to try the task and her/his behaviors were video recorded. This tube-using task ended either when the participant consumed all juice in the bottle or after one minute had elapsed. If participants left or looked away during the task, the experimenter attempted to attract their attention by calling the name, jiggling the tube, or adding more juice every 20 s. Another tube was provided if the participant moved away from the panel and left the previous tube beyond arm’s reach.

In each trial, one dipping-technique video and one sucking-technique video were shown in a pseudo-randomized order. Thus, each participant watched two videos per trial, and they participated in five trials in total, with an inter-trial interval of at least five days. A picture of nine identical icons (in a 3 × 3 grid) on a white background was displayed between the videos for attention check. Data collection continued if the participant’s attention was focused on the icons, as this indicated that they were still attending well to the stimuli.

### Analysis

**Fig. 1:**
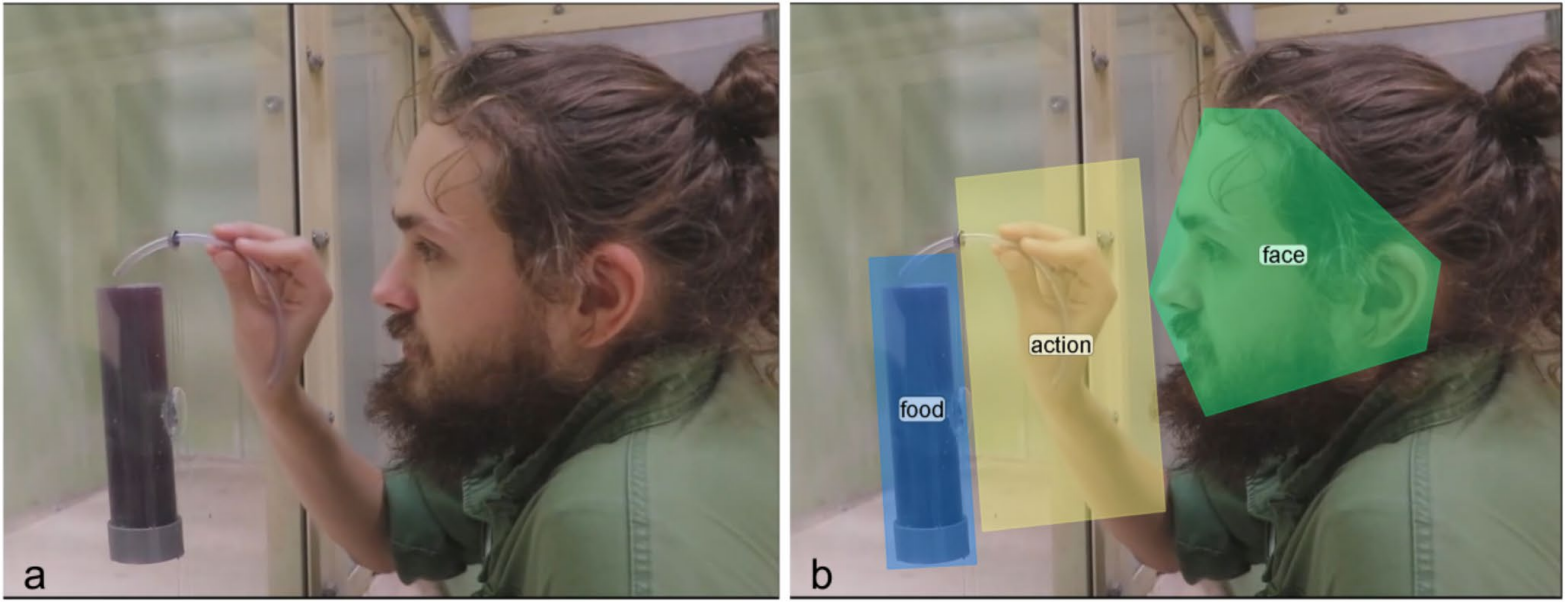
An example of video stimuli. An experimenter (J.B.) demonstrating the tube-using technique (**a**) and with main AOIs indicated (**b**)

Eye movement was filtered using Tobii Fixation Filter with default parameters. Area of interest (AOI) was defined for each video in the Tobii Studio software (ver. 3.2.1) and included the face (demonstrators’ face), food (juice bottle), action (tube in one hand), and screen (the whole screen) (Fig. 1). Statistical analyses were performed in R (v.4.3.2; R Core Team, 2024) using linear mixed effects models (LMM) (‘lmer’ in the package ‘lme4’) with Gaussian error structure and identity link function. The variable Trial was standardized to a mean of 0 and standard deviation of 1 (using the ‘standardize’ function) according to (Schielzeth 2010). We used the “check_model” function in the package “performance” to assess different aspects of a model’s fit by visual inspection of the diagnostic plots.

## Model: looking duration by knowledge*stimuli

We first investigated how participants with different tube-using knowledge viewed the videos. Total fixation duration was used for overall attention to the screen, and proportion of looking duration was used for each AOI (e.g. the proportion of looking duration to face is 0.5 if attention to face for 6 s with attention to the screen for 12 s). Separate models were run for each AOI. In all models, we included Trial (trial 1 to 5), Species (bonobos/chimpanzees), Demonstrator (first/second demonstrator), Knowledge (dipping/sucking), Stimuli (dipping/sucking videos), the interaction between Knowledge and Stimuli, and stimulus order (whether the first presented video was dipping/sucking) as fixed effects. We included participants’ identity (ID) to account for repeated measures for each individual, and the effects of Trial and Stimuli varying by ID as random effects and random slopes, respectively. The random-effects structure was kept maximal according to the suggestion of (Barr et al. 2013). The model syntax used for all models was: the proportion of Looking duration ~ Trial + Species + Demonstrator + Knowledge*Stimuli + First-show + (1 + Trial + Stimuli|| ID). We confirmed the normality of residuals, homogeneity of variance, and normality of random effects (ID) in all models. We also checked variance inflation factors (VIF) and found that collinearity remained low in all models (VIF < 5). When a significant interaction effect was detected in the model, we further examined it by testing simple effects within the subsets of data for each level of the predictors.

As exploratory analyses, we additionally tested whether the behavioral responses of participants indicated how they observed the stimuli and analyzed two smaller AOIs (eye and mouth) to investigate potential differences in participants’ attention. The modelling approach was similar to the above and results are reported in supplementary materials.

## Results

In total, all chimpanzees participated in 30 eye-tracking tests and 25 tube-using tasks in the testing room. Two individuals, Zamba and Misaki, were often eager to leave the room after eye-tracking and some of their tube-using tasks were therefore conducted in a room adjacent to the outdoor enclosure immediately upon their return to the enclosure. All bonobos completed the eye-tracking and tube-using tasks in their testing room. A naive observer coded 30% of the videos of tube-using tasks and showed high inter-rater reliability (Cohen’s Kappa test, Kappa value = 0.912, z = 6.2, *P* < 0.001) with the experimenter (Y.P.).

### Looking duration by knowledge*stimuli

**Fig. 2:**
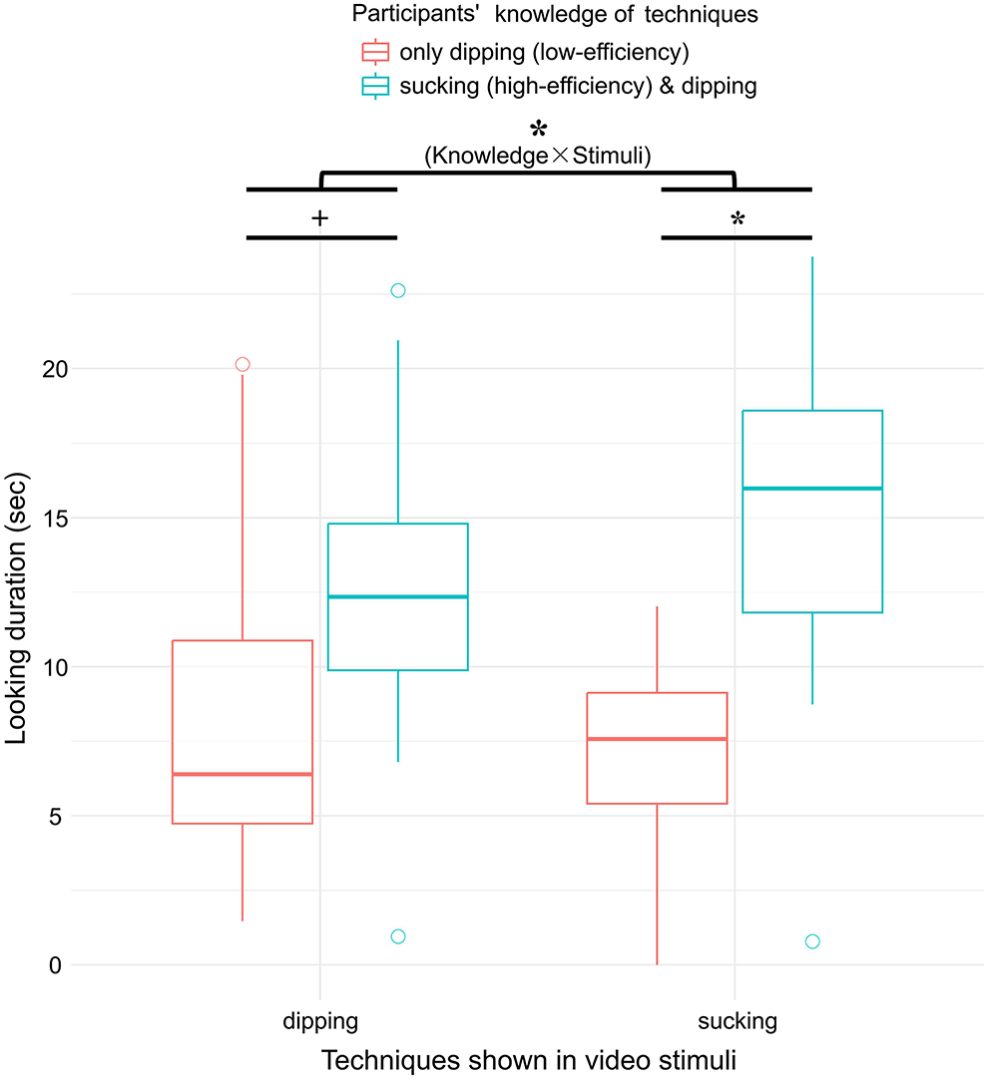
Looking duration to the screen by knowledge and stimuli.+ *P* < 0.1, * *P* < 0.05. All participants know the dipping-technique, but only sucking-technique participants know the more efficient method, i.e. sucking

A significant interaction between knowledge and video stimulus for overall attention to the screen was detected (β = 1.814, SE = 0.657, χ^2^ = 7.618, *P* = 0.020) (Fig. 2). We therefore tested simple effects in subsets of the data for each level of the test predictors. Sucking-technique participants looked significantly more at sucking-technique videos than dipping-technique participants (β = 4.245, SE = 1.340, χ^2^ = 10.037, *P* = 0.012). There was also a marginal effect of sucking-technique participants attending more to dipping-technique videos than dipping-technique participants (β = 2.871, SE = 1.284, χ^2^ = 4.997, *P* = 0.051).

The overall attention to the videos (whole screen) decreased from Trial 1 to 5 (β = − 0.696, SE = 0.147, χ^2^ = 22.382, *P* < 0.001). Similarly, participants looked less at the second demonstrator in the video (β = − 1.373, SE = 0.274, χ^2^ = 25.099, *P* < 0.001). There was no significant difference in the overall attention to the screen between species (χ^2^ = 0.116, *P* = 0.741), and the order of presenting videos did not significantly influence their looking duration (χ^2^ = 1.634, *P* = 0.206).

**Fig. 3:**
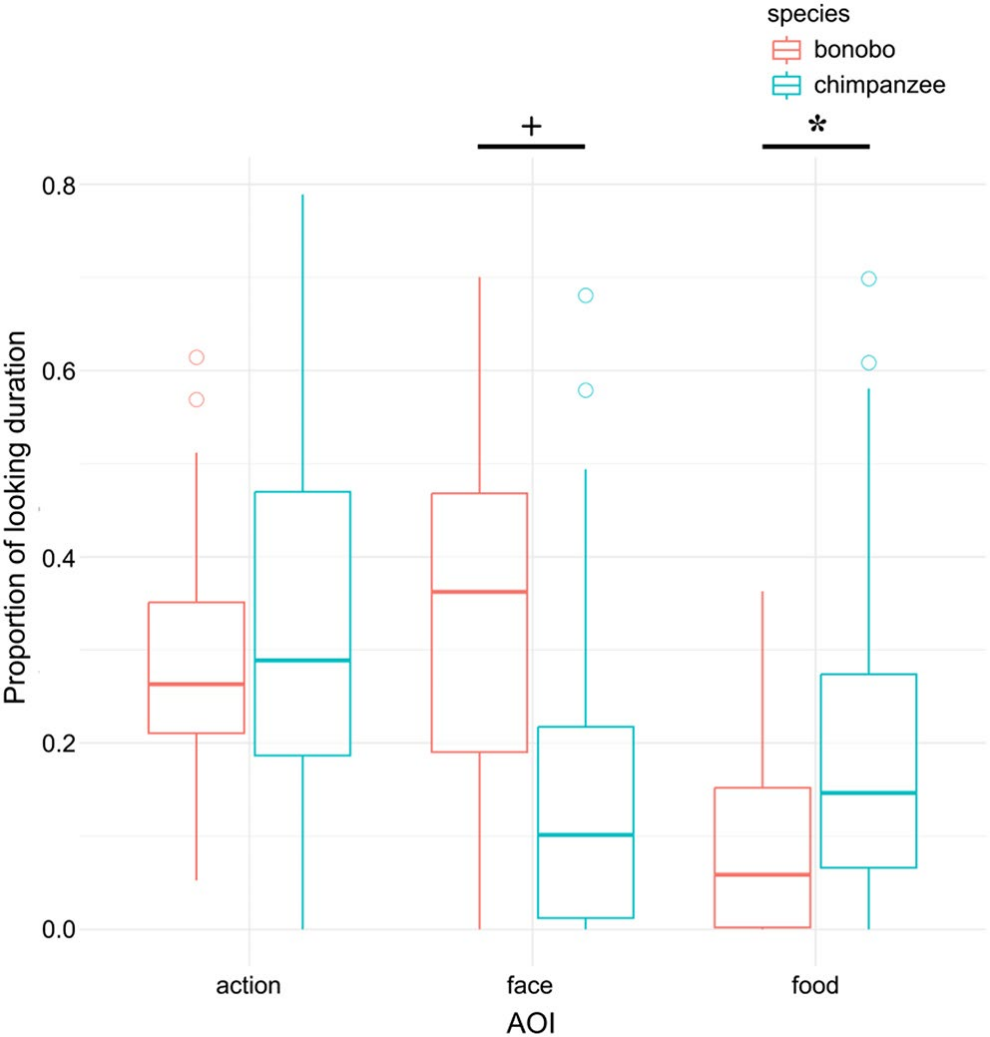
Proportion of looking duration to three AOIs in bonobos and chimpanzees. + *P* < 0.1, * *P* < 0.05

The proportion of looking duration to each AOI is shown in Fig. 3. Overall, the attention to the action AOI of both species did not show significant differences (χ^2^ = 0.868, *P* = 0.375). Specifically, it was not significantly influenced by trials (χ^2^ = 0.0005, *P* = 0.983), different demonstrators (χ^2^ = 0.496, *P* = 0.482), order of videos (χ^2^ = 0.0001, *P* = 0.994), tube-using knowledge (χ^2^ = 0.340, *P* = 0.574), or video stimuli (χ^2^ = 2.628, *P* = 0.116). For the face AOI, bonobos looked marginally longer compared to chimpanzees (β = − 0.192, SE = 0.093, χ^2^ = 4.237, *P* = 0.070). The trial number (χ^2^ = 3.074, *P* = 0.108), demonstrator (χ^2^ = 0.047, *P* = 0.828), video order (χ^2^ = 0.126, *P* = 0.723), tube-using knowledge (χ^2^ = 0.127, *P* = 0.730), and video stimuli (χ^2^ = 0.068, *P* = 0.799) did not have significant effects on participants’ attention to the face. For the food AOI, chimpanzees attended significantly more than bonobos (β = 0.138, SE = 0.057, χ^2^ = 5.908, *P* = 0.036), while the other predictors did not lead to any significant differences (Trial: χ^2^ = 0.252, *P* = 0.625, Demonstrator: χ^2^ = 0.593, *P* = 0.442, First-show: χ^2^ = 0.412, *P* = 0.522, Knowledge: χ^2^ = 0.303, *P* = 0.594, Stimuli: χ^2^ = 0.067, *P* = 0.799).

### Techniques employed following video demonstrations

Four of the six chimpanzees initially used the sucking technique while the other two used the dipping technique. All sucking-technique chimpanzees continued using this technique across the five trials, except for one individual (Hatsuka) who did not attempt the task at all in some trials. Neither of the dipping-technique participants attempted to use the sucking technique following demonstrations, and both showed low overall motivation to use the tool. All six bonobos initially used the dipping technique and none showed the sucking technique following video demonstrations. Surprisingly, only two individuals attempted to use the tool and employed the dipping technique during the test period, though others appeared interested in the juice by trying to use their fingers or tongue to reach it. Details and discussion of the coded behaviors of each participant can be found in supplementary materials.

## Discussion

This study used eye-tracking to test how chimpanzees and bonobos viewed tool-using demonstrations that were either the same as or different from their known techniques. We predicted that participants knowing different techniques would show different visual attention, and that success at learning the higher-efficiency sucking technique may differ between bonobos and chimpanzees (among those not already familiar with it). Results confirmed significant attentional differences to the screen and supported the first hypothesis, mainly driven by sucking-technique individuals looking significantly longer than dipping-technique individuals at sucking-technique videos, though also with a marginal difference in their attention to dipping-technique videos. Thus, dipping-technique participants did not closely attend to demonstrations of a technique that they did not know. The differences of their attention to the action, face, and food part was non-significant between dipping- and sucking-technique participants, indicating a similar attentional pattern to each AOI. As the species difference in the attention to the action part was not significant, the second hypothesis was not fully supported, though a tendency for bonobos to attend marginally more to faces was detected. None of the dipping-technique individuals of either species learned the sucking technique via the video demonstrations, so our third hypothesis was not supported.

While social learning from video demonstrations did not occur in our study, participants’ prior knowledge influenced their visual attention. Eye-tracking results showed that dipping-technique participants did not attend as closely to the videos, particularly the unfamiliar sucking technique, as those with prior knowledge of the technique. The motivation for the food between dipping and sucking participants may be a confound. However, we do not believe this can fully explain the results because most participants were motivated to drink the grape juice during the trials. Moreover, the participants have considerable experience participating in eye-tracking studies (Brooks et al. 2021; Kano and Hirata 2015; Kano et al. 2011, 2015, 2017) and generally show steady attention when viewing stimuli (Kano and Call 2017). In this study, both dipping and sucking participants showed good eye-tracking performance (indicated by “weighted gaze sample” in the Tobii Studio). Thus, it is unlikely that the observed differences are simply due to sucking-technique individuals having higher motivation or showing more attention to the stimuli. Close observation (peering) is a key factor for effective social learning (Schuppli et al. 2016; van Schaik 2010; Whiten and van de Waal 2018). As dipping-technique individuals did not closely attend to sucking videos, it is therefore unsurprising that they did not learn this technique from the demonstrations. It should be noted that all participants are familiar with the dipping technique (as they use sticks to dip for juice during regular environmental enrichment), but only sucking-technique individuals knew the high-efficiency method, i.e. sucking. Interestingly, although all participants sucked on the juice drip during eye-tracking, dipping-technique participants did not generalize this behavior to a tool-using context. Due to the lack of prior knowledge (Manrique and Call 2011), dipping-technique individuals only knew the low-efficiency method and likely did not understand the action of using the tube to suck. Thus, they may have been fixated on perceiving the tube solely as a dipping tool. Additionally, because of the interplay of action observing, understanding, and execution (Ferrari et al. 2009; Liepelt et al. 2008; Rizzolatti and Craighero 2004), individuals that lack an understanding of the action may additionally have little interest in observing it.

Notably, apes often maintain their interest in easily understandable content while unfamiliar and complex content, like animations and puppet plays, generally fail to keep their attention (Kano and Call 2017). Thus, apes may simply not pay much attention to what they do not know well. Given that the life history of captive apes involves many opaque tool-use behaviors of humans, participants may be generally unmotivated to attend closely to human demonstrations. In this case, even familiar human demonstrators may not be suited for social learning of tool use. Low attention to human actions that are not (initially) understood, as found here, may partly explain failures to socially learn from humans in some past studies (Buttelmann et al. 2008; Neadle et al. 2020), despite abundant evidence for conspecific social learning (Hopper et al. 2015; Vale et al. 2017; Whiten and van de Waal 2018). It remains unclear whether using conspecific demonstrators might facilitate better attention and promote potential social learning in this paradigm. Future studies using eye-tracking with conspecific demonstrators could offer a more complete understanding of how they allocate their attention during the social learning process. Additionally, building on the aforementioned findings, social learning of tool use in great apes may only occur within a limited zone (Tennie et al. 2020; Whiten et al. 2009), where the ability to understand the action significantly influences the likelihood of the individual observing and learning the behavior.

More broadly, several methodological factors may also explain why dipping-technique participants failed to learn the sucking technique. Firstly, participants might not have received sufficient exposure to the demonstrations. In a previous study using the same task (Yamamoto et al. 2013), dipping-technique chimpanzees underwent one to four 10-min trials with a conspecific demonstrator before adopting the sucking technique, while our participants only received 24-second video demonstrations in each trial. However, as trials progressed, the participants increasingly lost interest in the videos overall, so simply increasing exposure in this paradigm may not be sufficient. Second, non-human primates generally learn less effectively from video demonstrations compared with live demonstrations (Anderson et al. 2017; Hopper et al. 2015), though they can learn from videos in some contexts (Dindo et al. 2011; Gunhold et al. 2014; Price et al. 2009; Xu et al. 2024). Future work should also explore if there are any species differences in the ability to learn from videos between chimpanzees and bonobos, as well as other primates. In this study, two bonobos did seem to extract some information from video demonstrations (attempting to dip after ignoring the tool during familiarization trials in the testing room), though attention did not clearly differ in these trials and the sample size was too small to draw any conclusions. Third, demonstrator identity may be crucial. Both human demonstrators were familiar to apes for many months or years, but participants did look less at the second demonstrator (Y.P.), who they had known for a shorter time. This may be due to either demonstrator familiarity or participants quickly losing interest in repeated content. Future studies should explore these possibilities with more controlled conditions.

Our study used eye-tracking technology to investigate the social learning process of great apes, finding that they may not closely observe things that they do not know well (at least when performed by human demonstrators). This result provides potential explanations for some negative results in previous experiments and suggests the importance of using conspecific demonstrators in future studies. Although this study did encounter difficulty in distinguishing between the species and knowledge effects due to the small sample size, it also emphasizes the potential of applying eye-tracking to the study of social learning. With modified protocol and visual stimuli, such paradigms can also be used to study social learning in diverse species, providing insights into the foundational cognitive processes that underpin social learning and culture in non-human animals.

## Electronic supplementary material

Below is the link to the electronic supplementary material.

Supplementary Material 1

Supplementary Material 2

Supplementary Material 3

Supplementary Material 4

Supplementary Material 5

Supplementary Material 6

## Data Availability

Data is provided within the supplementary information files.

## Declarations

### Preprint policy

This manuscript (Piao et al. 2024) is posted on the preprint server, bioRxiv, on August 01, 2024. https://doi.org/10.1101/2024.08.01.605994.
