# Supplementary material for: An eye-tracking study of visual attention in chimpanzees and bonobos when viewing different tool-using techniques
Source: Anim Cogn. 2025 Feb 11;28(1):12. doi: 10.1007/s10071-025-01934-5 (PMC11814020; doi:10.1007/s10071-025-01934-5)
Supplement: Supplementary file 5 — Supplementary Material 5 [file 10071_2025_1934_MOESM5_ESM.docx]

Supplementary video

**Video S1** Examples of eye-tracking recordings. Participants watching human experimenters (Y.P & J.B.) demonstrating the two techniques.
